# Feasibility of combining a sensorimotor foot orthosis with a prefabricated carbon-fiber ankle-foot orthosis: A pilot study

**DOI:** 10.33137/cpoj.v9i1.47162

**Published:** 2026-05-10

**Authors:** Y. Aljohani, A. Alamri, G. Fiedler

**Affiliations:** 1 Department of Prosthetics and Orthotics, College of Medical Rehabilitation Sciences, Taibah University, Madinah, Saudi Arabia.; 2 King Abdulaziz University, Jeddah, Saudi Arabia.; 3 Department of Rehabilitation Science and Technology, School of Health and Rehabilitation Sciences, University of Pittsburgh, Pittsburgh, USA.

**Keywords:** Foot Orthosis, Sensorimotor Stimulation, Foot Drop, Orthosis, Stroke, Traumatic Brain Injury, Cerebral Palsy, Lower-Limb Orthoses, Ankle-Foot Orthosis, 2MWT, Sensorimotor

## Abstract

**BACKGROUND:**

Stroke, traumatic brain injury, cerebral palsy, and other neuromuscular impairments can result in foot drop, which reduces toe clearance, increases fall risk, and limits mobility. Ankle-foot orthoses (AFOs) are frequently prescribed to address foot drop. Sensorimotor foot orthoses (SMFOs) represent an alternative approach intended to influence muscle activation through sensory stimulation; however, evidence supporting their combined use with AFOs remains limited.

**OBJECTIVE:**

To evaluate the feasibility of a subsequent Randomized Controlled Trial (RCT) comparing a prefabricated carbon-fiber AFO worn with and without an SMFO in individuals with neuromuscular or musculoskeletal conditions.

**METHODOLOGY:**

A six-month randomized crossover pilot trial was conducted from February to July 2025 at the Department of Rehabilitation Science and Technology, University of Pittsburgh. Three participants with neuromuscular or musculoskeletal conditions were enrolled. The two conditions compared were a prefabricated carbon-fiber AFO (F5 AFO, Thrive Orthopedics, USA) worn alone and the same AFO worn in combination with a custom-made SMFO designed to activate muscles through exteroceptor and proprioceptor stimulation (Springer Aktiv, Germany). The primary outcome was average daily step count measured by a wearable pedometer. Secondary outcomes included gait performance using the Two-Minute Walk Test (2MWT), dynamic balance using the Narrowing Beam Walking Test (NBWT), mobility using the Rivermead Mobility Index (RMI), and device experience assessed via an Ecological Momentary Assessment (EMA) survey.

**FINDINGS:**

Three subjects enrolled; one withdrew due to difficulty finding footwear that accommodated the SMFO when worn in combination with the AFO. Two participants completed the study (mean age = 65 years), responded to approximately 77% of EMA prompts, and reported no adverse events. Step count was higher in the SMFO+AFO condition for one participant (1,952 vs. 116 steps) and comparable between conditions for the other (884 vs. 933 steps). Differences on the NBWT and RMI were minimal across conditions; RMI scores were at ceiling for both participants. On the 2MWT, Participant 1 walked 470 m with the AFO alone and 404 m with the combined condition, while Participant 2 walked 303 m and 294 m, respectively. EMA ratings for comfort, function, and satisfaction were higher, and inhibiting effects and wear time were lower with the combined condition throughout the study period.

**CONCLUSION:**

The protocol was feasible, safe, and acceptable to participants, with no adverse events and minimal participation burden. These findings support the design of a larger RCT to confirm these preliminary observations.

## INTRODUCTION

Neuromuscular impairments such as stroke, traumatic brain injury, cerebral palsy, peroneus paralysis, and other related conditions affect the functional abilities of patients.^1,2^

Globally, around 101 million individuals have survived a stroke, 69 million live with traumatic brain injury, and cerebral palsy occurs in approximately 2.1 per 1,000 live births.^3–5^ Foot drop (FD), which affects approximately 14% of stroke survivors as a long-term complication,^6,7^ results from weakness of the dorsiflexor muscles and leads to impaired toe clearance during the swing phase of gait, thereby increasing the risk of falls and reducing gait efficiency.^8^

Current medical approaches to address FD include physiotherapy, surgery, functional electrical stimulation (FES), and management by ankle-foot orthosis (AFO). Among these, AFOs are the most frequently prescribed interventions.^8^ They enhance the alignment of the ankle joint medio-laterally, prevent foot drop to improve toe clearance during the swing phase by reducing the need for compensatory movements and thereby decreasing walking energy consumption.^9^ However, the use of static AFOs may contribute to muscle atrophy over time due to restricted ankle range of motion.^10^

To counteract muscle atrophy and promote muscle activation, FES was developed to stimulate the nerves responsible for controlling the weak muscles by delivering small electrical impulses via electrodes on the skin.^11^ This technology can be effective but depends on proper timing and dosing of impulses and on battery power to operate. Sensorimotor stimulation represents another approach to muscle activation, but through a different mechanism. Its principle is to activate muscles indirectly by stimulating exteroceptors and proprioceptors. For example, Ludwig et al.^12^ demonstrated that sensorimotoric foot orthoses (SMFOs) stabilized the ankle joint by activating the peroneus longus muscle during mid-stance. Similarly, Simon et al.^13^ found that SMFOs reduced retropatellar stress in patients with patellofemoral pain.

Nevertheless, evidence on the use of SMFOs to improve clinical outcomes for individuals requiring AFO management remains limited. Recent studies have examined the effects of SMFOs on muscle activation in soldiers with pes planovalgus deformity^14^ and on balance outcomes in healthy adults.^15^ A pilot study tested the effects of SMFOs on subjective pain perception, measured using a visual analog scale, in 340 patients with various orthopedic conditions, and concluded that they may reduce pain, highlighting the need for further research through a Randomized Controlled Trial (RCT).^16^ Furthermore, a recent scoping review evaluating the effects of footwear, orthoses, and insoles on balance in older populations highlighted the need for high-quality evidence, such as RCTs, in this area.^17^

N-of-1 and single-case methodologies are increasingly acknowledged as rigorous and effective approaches to collect individualized pilot data, particularly when RCTs are not feasible.^18^ N-of-1 pilot trials have been successfully used to explore the feasibility of various treatments and interventions.^19–21^ A pilot study was needed to evaluate the feasibility of a subsequent RCT comparing a prefabricated carbon-fiber AFO worn with and without an SMFO in individuals with neuromuscular or musculoskeletal conditions. The hypothesis was that a crossover pilot protocol comparing a prefabricated carbon-fiber AFO worn in combination with a custom SMFO versus the AFO worn alone would be feasible to conduct.

## METHODOLOGY

### Study Design

**Figure 1:**
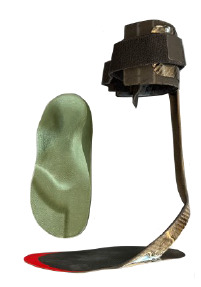
Prefabricated carbon-fiber AFO with posterior medial^22^ or lateral^23^ strut and custom sensorimotor foot orthosis (SMFO)^24^ used in the study.

**Figure 2:**
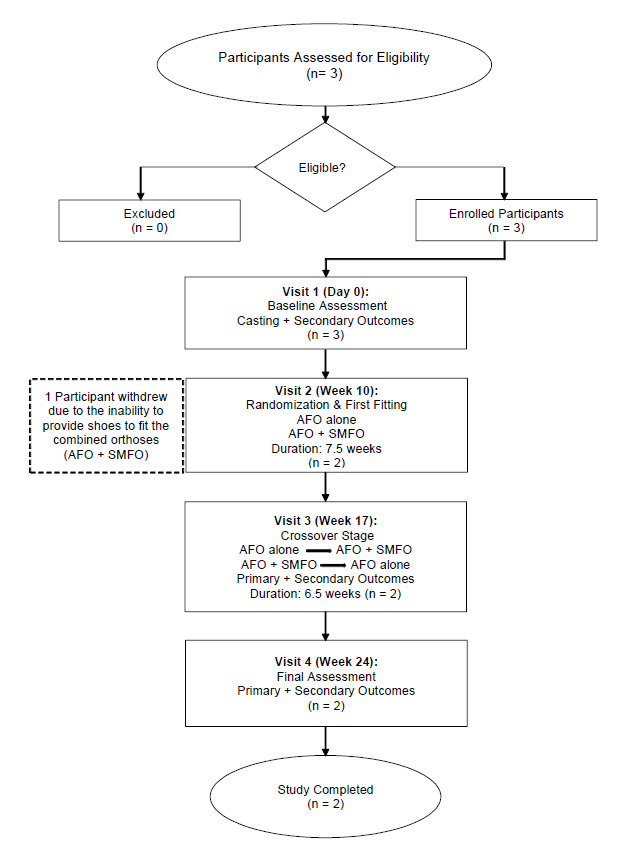
Participant flowchart illustrating enrollment, randomization, and study completion.

A randomized crossover pilot trial was conducted to compare two orthotic conditions. A prefabricated carbon-fiber AFO (F5 AFO with magnetic lock, Thrive Orthopedics, USA), with either a posterior medial^22^ or posterior lateral strut design^23^ as clinically indicated for each participant, was worn alone (**Figure 1**) or in combination with a custom-made sensorimotor foot orthosis (SMFO) (Proprio SOLE [Neuro], Springer Aktiv, Germany).^24^ The sequence of both conditions was randomized for each participant. The study was conducted at the university of Pittsburgh research laboratory over six months from February to July 2025 and consisted of four stages (**Figure 2**): baseline assessment and casting, first fitting, crossover to the alternate condition, and final assessment visit. Ethics review and approval was obtained from the University of Pittsburgh Institutional Review Board.

### Participants

Three participants with a history of lower-limb musculoskeletal or neurological conditions were recruited through Pitt+Me website page and enrolled in the study. All communications and study appointments arrangements were made by the study research team. Eligibility criteria included being 18 years of age or older, having a prescription for an AFO, and the ability to ambulate independently with shoes (with or without the orthosis). Exclusion criteria were recurrent stroke or other medical conditions that compromised walking ability or skin integrity. All participants provided written informed consent prior to enrolment.

### Outcome Measures

#### Primary outcome

The primary outcome was average daily step count, measured using a wearable pedometer (Yamax EX510 3D Power-Walker Pedometer). This device records and stores daily step counts for up to 30 days. For analysis, step counts from the 30 days immediately preceding each assessment visit were used.

#### Secondary outcomes

Walking distance was assessed with the Two-Minute Walk Test (2MWT), a valid and reliable measure of walking ability and physical capacity.^25–27^ The 2MWT is a suitable alternative to the Six Minute Walk Test (6MWT) or 12 Minute Walk Test for individuals with reduced walking endurance. Dynamic balance and postural control were tested using the Narrowing Beam Walking Test (NBWT), which has participants walk along a progressively narrowing path.^28–30^ Further distances walked on the NBWT indicate better balance ability and decreased fall risk. In this study, participants walked on the beam once, and the distance raw score was recorded. Mobility performance was recorded with the Rivermead Mobility Index (RMI), a 15-item questionnaire assessing a range of mobility tasks, from basic transfers to walking outdoors.^31,32^ Day-to-day feedback was collected using Ecological Momentary Assessment (EMA) surveys.^33^ Delivered two or three times daily at random times per text message and online surveying platform (Qualtrics, Provo, UT), these surveys captured device wear time, comfort, function, inhibiting effects, and overall satisfaction on 0–100 rating scales, along with contextual questions on activity, location, mood, and device attention.

### Assessment of Protocol Feasibility

Recruitment efficiency, protocol adherence, attrition, and adverse effects were documented to assess the feasibility of implementing this protocol in a larger-scale trial. The duration of appointments, effectiveness of data collection approaches, and unstructured participant feedback were also recorded throughout the trial.

### Intervention Procedures

Participants completed a baseline assessment of the secondary outcomes (2MWT, NBWT, and RMI) and were cast for individual customization. Bilateral casting was performed by a licensed clinician using fiberglass wraps under weight-bearing conditions to capture limb geometry in a functional position. The resulting casts were digitized using SnugFit O&P 3D scanner (Version 3.7.3; Xyken, LLC, McLean, VA, USA). The digital models and completed measurements documenting key anatomical dimensions and clinical specifications, including but not limited to (foot length, foot width, diagnosis, muscle status, and gait presentation) were sent to Springer Aktiv for fabrication of custom SMFOs. The proprio SOLE [Neuro] SMFOs are made from EVA foam with memory function, designed to offer targeted pressure point stimulation, cushioning, and vibration absorption (**Figure 1**).

At first fitting stage (duration of 7.5 weeks) one participant was fitted with the prefabricated carbon-fiber AFO alone and the other fitted with the AFO combined with the SMFO, and both received their pedometer devices to begin recording their step counts (fixed on the calf cuff of their AFOs). The AFO was a prefabricated carbon-fiber orthosis with either a posterior medial^22^ or posterior lateral strut design^23^ selected based on the clinical presentation of each participant (participant with PTTD was fitted with a posterior lateral strut, and participant with CVA was fitted with a posterior medial strut). The SMFO was individually fabricated based on sensorimotor principles.^12^ For the combined condition, the SMFO was positioned on top of the AFO footplate inside the shoe, such that the foot was in direct contact with the SMFO. All orthoses were fitted bilaterally by the same clinician and using standard fitting procedures.

At crossover stage (duration of 6.5 weeks) before switching to the next intervention, the pedometer device was reviewed to record step count for the past 30 days, and participants completed the second outcomes assessment. Collecting data only over the last 30 days of each intervention reduced the impact of the slight differences in intervention periods (dictated by scheduling constraints).

At the final assessment visit, step count was recorded, and participants underwent the last secondary outcomes assessment.

The first days of each intervention phase served as an adjustment and break-in period to allow participants to become accustomed to the new device by slowly increasing daily wear time. For the respective second intervention phase for each participant, this seven-to-fourteen-day time span also served as a wash-out period. Both participants and assessors were aware of the interventions sequence.

### Data Analysis

Descriptive statistics were conducted due to the small sample size. Outcomes were reviewed across time points and phases to assess feasibility and patterns between the two conditions. Effect sizes were calculated descriptively from the 232 repeated EMA observation pairs. Cohen's d was calculated as the mean difference divided by the standard deviation of differences for outcomes meeting parametric assumptions. For remaining outcomes, effect size r was derived from the Wilcoxon signed-rank test and converted to Cohen's d. Feasibility was assessed by documenting attrition, adverse events, appointment duration, EMA survey response rate, step count data completeness, and participant burden. The analysis was conducted using IBM SPSS Statistics for Windows, version 31.0 (IBM Corp., Armonk, NY, USA).

## RESULTS

Three subjects were enrolled in the study (**Figure 2**), of whom one withdrew after the first intervention period due to difficulty accommodating the SMFO within footwear when worn in combination with the AFO. The remaining two participants (mean age = 65 years) (**Table 1**) completed the study and reported an acceptable burden of participation and no complaints. During the study, no adverse events or disruptions occurred. Average appointment duration did not substantially deviate from the allocated 75 minutes for the intake appointment and 30 minutes for follow-ups. EMA survey response rate was approximately 77% (not counting the dropped-out participant after the withdrawal), resulting in a total of 232 data points for this part of the study. There were no noticeable gaps in the step count data. Step count was higher with the SMFO+AFO condition for Participant 1 (1,952 vs. 116 steps) and comparable between conditions for Participant 2 (884 vs. 933 steps).

**Table 1:** Participant characteristics (N = 2), Posterior Tibial Tendon Dysfunction (PTTD), Cerebral Vascular Accident (CVA), intramedullary (IM).

Characteristic	Participant 1	Participant 2
**Age (years)**	62	68
**Gender**	Female	Female
**Height (cm)**	155	168
**Weight (kg)**	115	68
**Diagnosis**	PTTD	CVA
**Other medical conditions**	Varicose veins	Right femur IM Nail, hammer toe, equino varus
**Date of diagnosis**	2019	2019
**Affected side**	Right	Right
**Mobility aid**	None	Cane
Activity level*	Light	Light

* The activity level reflects a subjective classification provided by the participant and is not based on a standardized scale.

**Figure 3:**
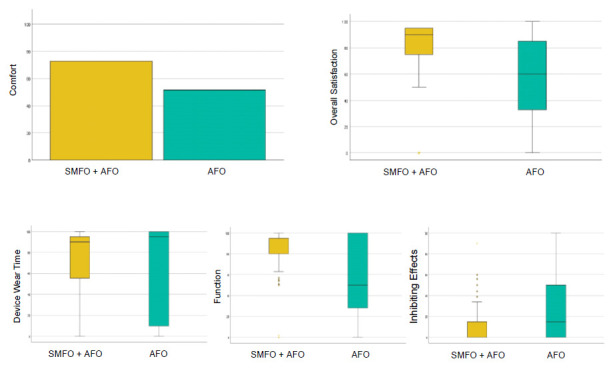
EMA ratings for comfort, overall satisfaction, function, device wear time, and inhibiting effects comparing the SMFO combined with prefabricated carbon-fiber AFO and the prefabricated carbon-fiber AFO alone. Comfort is displayed as a bar graph (mean); remaining outcomes are displayed as box plots (median, IQR).

Differences on the NBWT and RMI were minimal across conditions. On the 2MWT, Participant 1 walked 470 m with the AFO alone and 404 m with the combined condition; Participant 2 walked 303 m and 294 m, respectively. The assessments during the site visits were efficient, but a ceiling effect was observed in the RMI survey (**Table 2**). Descriptive outcomes are reported in **Table 3** and **Figure 3**. On average, the descriptive results showed that ratings during the SMFO condition for comfort, function, and satisfaction were higher compared to the prefabricated carbon-fiber AFO alone. Inhibiting effects and wear time ratings were lower with SMFO use (**Table 3**). The largest effect was observed for the device wear time (d = −0.49) while other outcomes showed only small differences between the two conditions (**Table 3**).

**Table 2:** Functional outcomes comparing the prefabricated carbon-fiber AFO worn in combination with the SMFO and the prefabricated carbon-fiber AFO worn alone across participants.

Outcome	Participant 1			Participant 2		
	Baseline	SMFO + AFO	AFO	Baseline	SMFO + AFO	AFO
Average Step count	_	1,952	116	_	884	933
2MWT (m)	477	404	470	249	294	303
NBWT (m)	3.7	3.7	3.4	0.0	0.3	0.0
RMI	15	14	15	12	13	12

2MWT = Two-Minute Walk Test; NBWT = Narrowing Beam Walking Test; RMI = Rivermead Mobility Index.

**Table 3:** Mean (SD), median, and effect size estimates of EMA outcomes.

Outcome	AFO + SMFO Mean ± SD (Median, Range)	AFO Mean ± SD (Median, Range)	Effect Size
Device Wear Time	73.5 ± 32.3 (90, 0–100)	61.7 ± 42.9 (95, 0–100)	d = − 0.49
Comfort	75.1 ± 29.4 (90, 0–100)	53.0 ± 33.2 (50, 0–100)	d = 0.04
Function	79.9 ± 29.5 (95, 0–100)	53.6 ± 37.2 (50, 0–100)	d = − 0.14
Inhibiting Effects	9.6 ± 15.1 (0, 0–90)	25.2 ± 25.8 (15, 0–100)	d = − 0.12
Overall Satisfaction	78.0 ± 27.5 (90, 0–95)	55.0 ± 33.4 (60, 0–100)	d = − 0.02

All outcomes, including device wear time, were based on subjective ratings from participants recorded on 0–100 scales. Means and SDs are based on repeated EMA survey responses collected throughout the study period. N = 232 responses. Function, overall satisfaction, device wear time, and inhibiting effects were non-normally distributed and analyzed using the Wilcoxon signed-rank test; Comfort was analyzed using a paired t-test. Effect sizes are reported as Cohen’s d for all outcomes.

## DISCUSSION

The main goal of this study was to establish the feasibility of a clinical trial protocol to investigate medium-term outcomes of an intervention that combines AFO and SMFO use in people with neuro-muscular lower limb impairments that affect gait. This approach is commonly utilized in the early stages of trial development, including in the field of orthotics, before a larger sample is subjected to a research intervention.^34,35^

This pilot study demonstrated that the research protocol was feasible, safe, and acceptable to participants. No adverse events occurred, and participation burden was minimal. This supported the assumption that combining two interventions whose independent safety and tolerability has been established previously does not cause undesired interaction effects.^36–38^ Appointments were completed within typical clinical durations (60 to 90 minutes for initial fitting, less than 30 minutes for follow up appointments), and were comparable to standard AFO care. Based on these insights, the SMFO appears practical for clinical settings. One subject withdrew due to difficulty fitting footwear, which highlights a practical consideration for future trials, but also aligns with experiences from similar longer-duration trials where retention rates of around 75% were reported.^39,40^

Preliminary outcomes suggested that adding a SMFO to a prefabricated carbon-fiber AFO may provide advantages over the AFO alone. Comfort, function, and satisfaction were higher, and inhibiting effects and wear time ratings were lower with SMFO use. This aligns with previously described effects of the SMFO intervention, of better muscle activation and lower pain.^12–16^ If confirmed in a properly powered clinical trial, these effects would translate into improved quality of life and participation for the targeted patient population.^41^ Effect size estimates provide useful information for planning such a trial. The largest effect was observed for wear time (Cohen’s d = −0.49), supporting its use as a primary endpoint. The effect size is comparable in magnitude to standardized mean differences reported in similar pilot studies, although direct comparison is limited by differences in study design and effect size definitions.^42,43^ The wash-out design appeared adequate based on a trend-analysis of the major outcome variables, as outcome patterns stabilized across periods, suggesting minimal carryover effects.

These findings should be interpreted with caution, as generalizability of any insights was limited by its very small and heterogeneous sample. A larger pilot trial, informed by the findings about protocol feasibility, should be conducted to arrive at dependable effect size estimations. The possibility of carryover effects between conditions could not be formally assessed given the small sample size. The primary outcome of daily step count reflects overall activity but does not directly capture biomechanical or qualitative aspects of gait, which may respond differently to the intervention. Although EMA compliance was acceptable, incomplete responses may introduce bias if missingness was related to user experience. Blinding was not feasible due to the nature of the intervention, which may have influenced subjective outcomes such as comfort and satisfaction. Nevertheless, the data are valuable for refining study procedures, addressing logistical challenges, and informing sample size calculations for future research.

In a planned larger scale study, much of the tested protocol should be adopted. The burden of participation for participants is acceptable, the logistics of the protocol administration are manageable, and the costs of the study intervention are low enough to be compatible with a typical research budget in the field. Only the survey-based outcome tool (Rivermead Mobility Index) showed ceiling effects and should be replaced with a different option, for instance the High-Level Mobility Assessment Tool.^44^

## CONCLUSION

This pilot randomized crossover study demonstrated that the protocol comparing a prefabricated carbon-fiber AFO worn in combination with a custom SMFO versus the AFO worn alone in individuals with lower-limb impairments was feasible to conduct in a clinical research setting, supporting the development of a larger-scale randomized controlled trial.
